# Supplementary material for: Association of MG53 with presence of type 2 diabetes mellitus, glycemic control, and diabetic complications
Source: PLoS One. 2023 Sep 12;18(9):e0291333. doi: 10.1371/journal.pone.0291333 (PMC10497120; doi:10.1371/journal.pone.0291333)
Supplement: Attachment — Submitted filename: Response to Reviewers.docx [file pone.0291333.s002.docx]

June 16, 2023

Dear Editor and Reviewers,

We would like to thank you for your comments, suggestions, and effort in the article. Your correction suggestions have been thoroughly reviewed by all our authors. We would like to state that we agree with your suggestions and criticisms and that your suggested revisions add value to our article. Necessary arrangements have been made in this direction. The revisions made are detailed below, and the current version of the article has been uploaded to the system.

**Modifications on reviewer 1's specific recommendations:**

1- It has been added to the discussion in detail that the study findings cannot be universally generalized because the study design and population did not meet the external validity, only included participants of Turkish ethnic origin, and insulin resistance in the control group could not be excluded entirely.

*On page 18, lines 272-280: ‘’* When interpreting the results of our study, it should be considered that it was single-centered and performed only in the Turkish ethnic group. There may be an association between MG53 and insulin resistance and diabetes in other ethnic populations elsewhere. Furthermore, given the high prevalence of insulin resistance in Turkey and the lack of comprehensive exclusion of insulin resistance in the studied population, it is challenging to generalize the results of this study. Moreover, there are limited clinical studies and, in particular, multicenter clinical randomized trials or systematic reviews to build solid evidence for this conclusion. The imperative finding of this study cannot be generalized to other populations because the study methods (design, setting, and population) do not meet external validity. The design of our study cannot refute previous evidence but rather provides clinical insight.’’

2- The conclusion part has been updated in line with your suggestion.

*On page 18-19, lines 283-286: ‘’* Our research has shown that there is no significant connection between the levels of circulating MG53 and T2 DM or glycemic control in the studied population of Turkish ethnicity. However, we believe that further clinical studies should be conducted to establish a more solid link between MG53, insulin resistance, and glycemic control across a broader range of populations worldwide.’’

3- In the abstract, the conclusion part has been rewritten as you stated, with the term " therapeutics targeting MG53" removed.

*On page 2, lines 40-43: ‘’* Our research finding provides insightful clinical evidence of lack of association between the levels of MG53 and T2 DM or glycemic control, at least in the studied population of Turkeys ethnicity. However, further clinical studies are warranted to establish solid evidence of the link between MG53 insulin resistance and glycemic control in a wider population elsewhere in the world.’’

4- Your suggestions regarding Exclusion Criteria and study limitations have been detailed in the limitations section and have been rearranged.

*On page 19, lines 293-295:’’* Finally, the control group consisted of healthy individuals who did not meet the clinical and demographic parameters associated with insulin resistance. However, the lack of HOMA-IR index data may suggest that the presence of insulin resistance in the control group could not be excluded entirely.’’

**Other editorial edits:**

1- The article has been checked for compliance with the style requirements of PLOS ONE, including for file naming.
2- Acknowledgments Section has been rearranged as suggested.

*On page 19, lines 296-297: ‘’* We would like to express our deep and sincere gratitude to our research supervisor, Dr. S. Guldiken, Endocrinology and Metabolism.’’

3- The full ethics statement has been added to the "Methods" section of our article file.

*On page 4, lines 83-84: ‘’* The Ethics Committee of Trakya University approval was granted before the study (TUTF-BAEK 2021/414).’’

4- The reference list has been reviewed to ensure it is complete and accurate.

Kind Regards,

Burak Andac, MD
